# Dopamine Increases the Intrinsic Excitability of Parvalbumin-Expressing Fast-Spiking Cells in the Piriform Cortex

**DOI:** 10.3389/fncel.2022.919092

**Published:** 2022-06-09

**Authors:** Yasmin Potts, John M. Bekkers

**Affiliations:** Eccles Institute of Neuroscience, John Curtin School of Medical Research, The Australian National University, Canberra, ACT, Australia

**Keywords:** action potential, neuromodulation, interneuron, olfaction, Parkinson’s disease

## Abstract

The piriform cortex (PCx) is essential for the adaptive processing of olfactory information. Neuromodulatory systems, including those utilizing serotonin, acetylcholine, noradrenaline, and dopamine, innervate and regulate neuronal activity in the PCx. Previous research has demonstrated the importance of acetylcholine, noradrenaline and serotonin in odor learning and memory. In contrast, the role of dopamine in the PCx remains under-explored. Here we examined how dopamine modulates the intrinsic electrical properties of identified classes of neurons in the PCx. We found that dopamine had no consistent effect on the intrinsic electrical properties of two types of glutamatergic neurons (semilunar and superficial pyramidal cells) or three types of GABAergic interneurons (horizontal, neurogliaform and somatastatin-expressing regular-spiking cells). However, dopamine had a striking effect on the intrinsic excitability of the parvalbumin-expressing fast-spiking (FS) class of GABAergic interneuron. Dopamine depolarized the resting potential, increased the input resistance and increased the firing frequency of FS cells. Co-application of dopamine with the D1-class dopamine receptor antagonist SCH 23390 blocked the effects of dopamine modulation on FS cells. Conversely, co-application of dopamine with the D2-class antagonist RS-(±)-sulpiride had no effect on dopamine modulation of these cells. Our results indicate that dopamine binds to D1-class dopamine receptors to increase the intrinsic excitability of FS cells. These findings suggest that dopamine has a highly targeted effect in the PCx and reveal how dopamine may modulate the balance between excitation and inhibition, with consequences for odor processing. In addition, our findings provide clues for understanding why neurodegenerative disorders that modify the dopamine system, such as Parkinson’s disease, have a deleterious effect on the sense of smell, and may suggest novel diagnostics for the early detection of such disorders.

## Introduction

The piriform cortex (PCx) is a trilaminar paleocortex which processes and encodes olfactory information. The PCx receives the bulk of its afferent input from the olfactory bulb while showing extensive synaptic connectivity with other brain regions, including the anterior olfactory nucleus, amygdala and orbitofrontal cortex (Majak et al., 2004; Illig, 2005; Hagiwara et al., 2012). The PCx also receives input from multiple neuromodulatory systems (Linster and Cleland, 2016). Previous research on neuromodulation in the PCx has shown that serotonin inhibits glutamatergic neurons (Lottem et al., 2016; Wang et al., 2020) while both noradrenaline and acetylcholine have been found to enhance long-term potentiation of synaptic transmission (Hasselmo and Barkai, 1995; Morrison et al., 2013). In contrast, the role of dopamine in the PCx is much less clear.

Dopamine has traditionally been viewed as a key contributor to the sensations of reward and pleasure (Schultz, 1998). However, more recent research has demonstrated the functional heterogeneity of dopamine signaling through its implication in arousal, motivational salience and memory (Ben Zion et al., 2006; Salamone and Correa, 2012; Puig et al., 2014). This functional heterogeneity, in conjunction with the vast dopamine projections throughout the brain (Aransay et al., 2015; Stagkourakis et al., 2019), means that dopamine dysfunction can result in numerous symptoms of Parkinson’s disease (PD) (Dauer and Przedborski, 2003), Huntington’s disease (Cepeda et al., 2014), attention deficit hyperactivity disorder (Volkow et al., 2009) and schizophrenia (Kesby et al., 2018).

Interestingly, the motor symptoms of PD, caused by a severe reduction of dopaminergic neurons in the substantia nigra, can be preceded by a declining sense of smell which can occur years earlier in up to 90% of patients (Fullard et al., 2017; Schapira et al., 2017). Dopamine projections along the mesocortical pathway are received by dopamine receptors in the PCx (Santana et al., 2008), suggesting that dopamine may modulate neural activity in this cortical structure. Prior research has found that dopamine increases spontaneous inhibitory activity in the PCx (Gellman and Aghajanian, 1993). Dopamine has also been reported to both decrease (Collins et al., 1985) and have little effect on (Schärer et al., 2012) excitatory activity in the PCx. In addition, changes in the olfactory bulb in animal models of PD have been described (Doty, 2012), suggesting that hyposmia in PD may also involve circuits earlier in the olfactory pathway.

To clarify previous findings and further explore dopaminergic effects at a cellular level, we have utilized single-cell electrophysiological techniques to examine how dopamine influences the intrinsic electrical properties of six cell types in the PCx. We found that dopamine had little or no effect on the intrinsic electrical properties of the glutamate-releasing semilunar and superficial pyramidal cells or the GABA-releasing horizontal, neurogliaform and regular-spiking cells. However, dopamine had a striking effect on the intrinsic electrical properties of the GABAergic fast-spiking (FS) interneurons. Our results demonstrate that dopamine acts on D1-class dopamine receptors to depolarize the resting potential, increase the input resistance and increase the intrinsic excitability of FS cells. Thus, dopamine acts on FS cells to modulate the delicate balance between excitation and inhibition in the PCx.

## Materials and Methods

### Slice Preparation

Slices were prepared using standard methods, as previously described (Suzuki and Bekkers, 2006), from postnatal day 18 to 25 heterozygous GAD67-GFP (△ neo) and PV-Cre-tdTomato transgenic mice of either sex bred on a C57BL6 background (Tamamaki et al., 2003; Kaiser et al., 2015). All animal housing, breeding, handling and surgical procedures were approved by the Animal Experimentation Ethics Committee of the Australian National University and conform to the guidelines of the National Health and Medical Research Council of Australia. Briefly, mice were anesthetized with 2% isoflurane in oxygen and quickly decapitated. The brain was removed and placed in an ice-cold cutting solution (in mM: 87 NaCl, 0.25 CaCl_2_, 3 MgCl_2_, 25 NaHCO_3_, 1.25 NaH_2_PO_4_, 75 sucrose, 25 glucose) bubbled continuously with 95% O_2_/5% CO_2_. Coronal slices (300 μm thick) of the anterior PCx were cut and incubated for 45 min at 35°C in artificial cerebrospinal fluid (ACSF; in mM: 125 NaCl, 3 KCl, 2 CaCl_2_, 1 MgCl_2_, 25 NaHCO_3_, 1.25 NaH_2_PO_4_, 25 glucose, 1 (+)-sodium L-ascorbate, 4 sodium pyruvate). Slices were subsequently held at room temperature until required.

### Electrophysiology

Whole-cell patch clamp techniques were used to measure electrical properties of neurons in the PCx, as described previously (Suzuki and Bekkers, 2006). Recordings were obtained *in vitro* using a top-focusing microscope equipped with differential interference contrast and fluorescence (Axioskop 2 FS, Zeiss, Germany). Coronal slices were placed into the microscope chamber and constantly perfused with ACSF, which was continuously bubbled with 95% O_2_/5% CO_2_ and maintained at 34 ± 2°C. Patch electrodes had resistances of 4–7 MΩ when filled with an internal solution (in mM: 135 KMeSO_4_, 4 KCl, 1 NaCl, 10 HEPES, 0.1 EGTA, 5 Phosphocreatine Na_2_, 2 Mg_2_ ATP, 1 Na_3_ GTP, 10 D-sorbitol, 0.4% biocytin, KOH for pH 7.2).

Neurons were selected for recording based on the layer in which their soma was located (layer 1, 2 or 3) and their expression of GFP in slices from GAD67-GFP (△ neo) mice (Tamamaki et al., 2003) (GFP^+^ for GABAergic interneurons, GFP^–^ for glutamatergic neurons). In some experiments FS cells were identified by their expression of tdTomato in slices from PV-Cre-tdTomato mice (Kaiser et al., 2015). Electrical recordings were made using a MultiClamp 700B amplifier (Molecular Devices, United States). During current clamp recordings, the pipette capacitance was compensated and the bridge balance was adjusted and monitored regularly. Data were sampled at 50 kHz by an ITC-18 digitizing interface (Instrutech/HEKA, Germany) under the control of AxoGraph (AxoGraph Scientific, Sydney).

In order to monitor action potential frequency, a depolarizing current step (1 s duration) was applied under the control condition and the current amplitude was adjusted to obtain a mean firing frequency of 5–10 Hz, depending on the cell type. The current amplitude was then kept constant for the remainder of the experiment. In some experiments the current amplitude was increased with a constant increment (40 pA) in order to measure firing frequency vs. current amplitude.

### Pharmacology

All drugs were bath-applied. Stock solutions (10 mM) were prepared for dopamine (Merck/Sigma-Aldrich, United States) and the D1- and D2-class dopamine receptor antagonists, SCH 23390 hydrochloride and (RS)-(±)-sulpiride (Hello Bio, United Kingdom). Stock solutions were stored at –20°C and thawed immediately before use. The stocks were diluted 1,000 × into ACSF, giving a final bath concentration of 10 μM.

### Morphology

Following recording, slices were fixed with 4% paraformaldehyde for 2–3 h, washed in phosphate buffered saline (PBS) and stored for up to 3 weeks in PBS at 4°C. Fills were visualized using streptavidin-Alexa Fluor-594 (Thermo Fisher Scientific/Invitrogen, United States) following the manufacturer’s instructions. Slices were imaged on a confocal microscope (Nikon A1, Nikon, Japan).

### Data Analysis

Passive intrinsic electrical properties, including input resistance and resting potential, were measured using AxoGraph. Active intrinsic electrical properties were calculated using custom Python code running under Jupyter Notebook (version 3.7.12). The firing frequency was defined as the number of action potentials (APs) per second which surpassed 0 mV. Individual AP data were collated to form an average AP for each cell under each condition (control, dopamine, wash). The AP peak amplitude and afterhyperpolarization (AHP) amplitude were defined as the maxima and minima of the average AP while the voltage threshold was defined as the first point where the derivative of the AP trace exceeded 20 V/s. The AP height was calculated by subtracting the AP voltage threshold from the AP peak amplitude. AP half-width was measured at 50% of the AP height.

### Statistical Analysis

Statistical analyses were done using R running under R studio (version 1.4.1106). Although data collation and analysis were not randomized or blinded, we made efforts to automate analysis whenever possible. Two-sample comparisons used Welch’s paired or unpaired 2-tailed *t*-test (*t*.test function in R). Multiple comparisons employed repeated measures ANOVA implemented using a linear mixed effects model with Tukey’s contrasts (*lmer, anova*, and *emmeans* functions in R) while taking care to correctly incorporate fixed and random effects. All statistical tests were applied to the raw data, whereas Figures 2C, 3B,C show normalized changes in parameters for display purposes only. These changes were calculated as described in the figure legends. Results are presented as mean ± standard error of the mean (SEM) unless stated otherwise, with the associated *p* and *n* values, where *n* is the number of cells. Statistical significance is denoted in the figures by one or more asterisks, following the convention in R, or by *ns* (not significant).

## Results

### Six Types of Neurons in the Piriform Cortex Can Be Differentiated by Their Soma Location and Electrophysiological Properties

**FIGURE 1:**
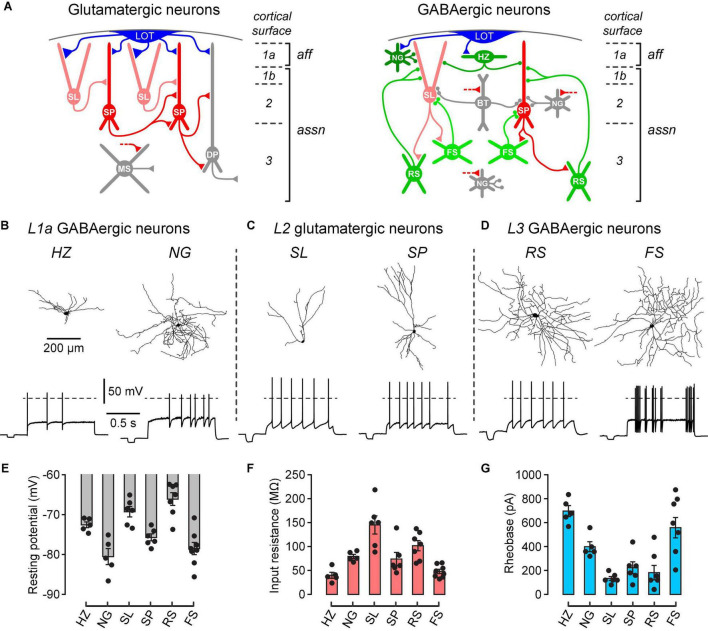
Different types of neurons in the PCx can be differentiated by their laminar location, dendritic morphology and electrophysiological properties. **(A)** Schematic diagrams showing the main classes of glutamate-releasing excitatory neurons (red and gray, left) and GABA-releasing inhibitory interneurons (green and gray, right) in the PCx. A selection of inferred synaptic connections is also shown. Gray cells were not examined in this study. LOT, lateral olfactory tract; SL, semilunar; SP, superficial pyramidal; DP, deep pyramidal; MS, multipolar spiny; NG, neurogliaform; HZ, horizontal; BT, bitufted; RS, regular-spiking; FS, fast-spiking; *aff*, afferent layer; *assn*, associational layers. Modified from Bekkers and Suzuki (2013), used with permission. **(B)** Morphological and electrophysiological properties of GABAergic HZ and NG neurons in layer 1a (*L1a*) of the PCx. Reconstructed dendrites (top) and typical firing patterns just above rheobase (bottom) of an HZ cell (left) and an NG cell (right). Dashed horizontal lines represent 0 mV. Scale bars apply to the corresponding panels for all cell types. **(C,D)** Similar data for SL and SP glutamatergic neurons in layer 2, and RS and FS GABAergic neurons in layer 3, respectively. **(E–G)** Bar plots showing the mean resting potential, input resistance and rheobase (± SEM), respectively, for the 6 types of neurons shown above. Filled symbols are measurements from individual neurons. The *n*-values are: HZ, 5; NG, 5; SL, 6; SP, 6; RS, 7; FS, 8.

In order to study the effect of dopamine on known types of neurons in the PCx, we first needed to confirm correct identification of these types. Figure 1A shows schematically the main types of glutamatergic excitatory neurons and GABAergic inhibitory interneurons in the PCx, as well as their synaptic connections and laminar locations. Layer 1a exclusively receives the afferent (*aff*) inputs from the olfactory bulb *via* the lateral olfactory tract (LOT), whereas layers 1b, 2 and 3 contain intracortical associational (*assn*) inputs (Figure 1A). Neuron identification was initially done by noting the layer in which the soma was located and the presence or absence of somatic fluorescence. GABAergic interneurons selectively express GFP in GAD67-GFP (△ neo) mice (Tamamaki et al., 2003), while FS interneurons selectively express tdTomato in PV-Cre-tdTomato mice (Kaiser et al., 2015). Most experiments used GAD67-GFP (△ neo) mice but some used PV-Cre-tdTomato mice to confirm the identification of FS neurons. Standard whole-cell recordings with biocytin in the intracellular solution were then used to measure the intrinsic electrical properties and recover the dendritic morphology of single neurons.

**FIGURE 2:**
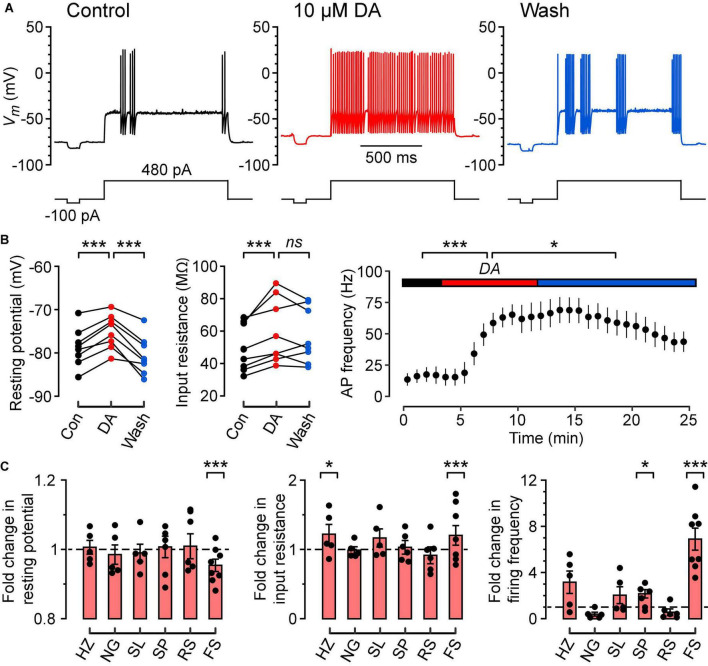
Dopamine depolarizes the resting potential, increases the input resistance and increases the frequency of firing of FS cells in response to a constant suprathreshold current step. **(A)** Typical action potential firing patterns of an FS cell during control (black), dopamine (DA) (red) and wash (blue) in response to a constant 480 pA current step. A separate current step (-100 pA) was also injected to monitor input resistance. **(B)** (Left), Resting potentials of FS cells (*n* = 8) in control, DA and wash; lines connect data points for each cell. **(B)** (Middle), Input resistances measured for the same FS cells. **(B)** (Right), Plot of mean action potential frequency vs. time for the same FS cells. Colored horizontal bars indicate the control, DA and wash periods [same color code as in **(A)**]. **(C)** Fold changes in resting potential (left), input resistance (middle) and firing frequency (right) caused by 10 μM DA, calculated by normalizing each parameter in DA to the mean of the corresponding parameters measured in control conditions, for HZ (*n* = 5), NG (*n* = 5), SL (*n* = 5), SP (*n* = 6), RS (*n* = 7), and FS cells (*n* = 8). Filled symbols are measurements from individual neurons. Bar plots and error bars indicate the mean ± SEM. The largest and most consistent effects of DA are seen in FS cells. ***p < 0.001; *p < 0.05; *ns*, not significant.

**FIGURE 3:**
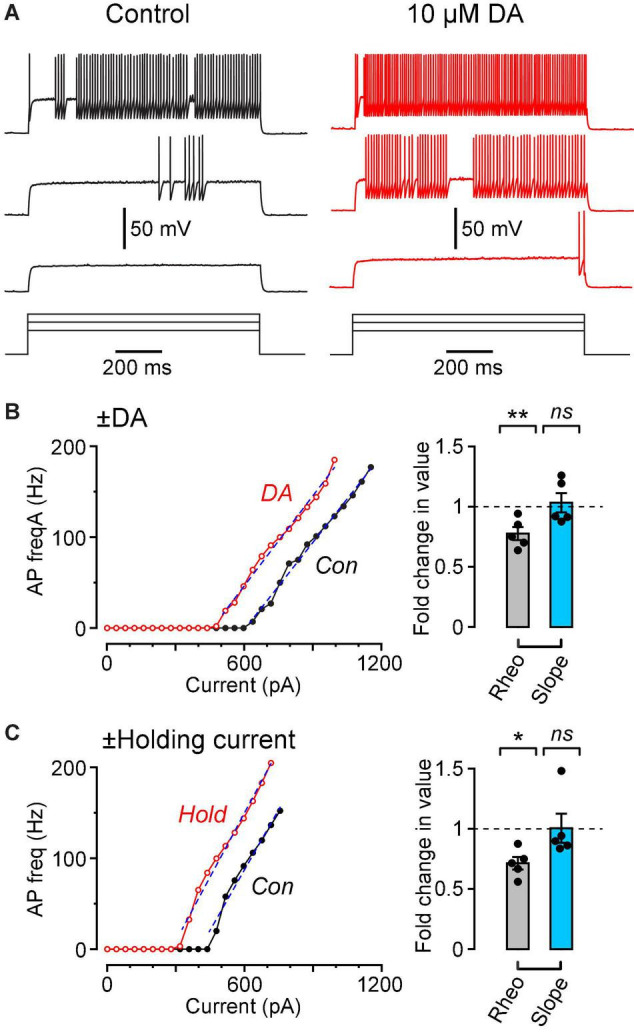
Dopamine causes a leftward shift in the f-I plot with no change in slope, similar to the effect of injecting a steady holding current. **(A)** *V*_*m*_ traces recorded in response to three 1 s-long current steps (480, 640, 800 pA) in the same FS cell in control (black traces, left) and in 10 μM DA (red traces, right). **(B)** (Left), Plot of AP frequency vs. injected current (f-I plot) for the same cell shown in **(A)**. Superimposed dashed lines are linear fits over the indicated ranges. **(B)** (Right), Summary plot showing the fold change in rheobase (‘Rheo’) and slope of the f-I plot (‘Slope’) caused by 10 μM DA, calculated by dividing the value for each cell in DA by the corresponding value for that cell in control conditions. Filled symbols are measurements from individual neurons (*n* = 5 FS cells). Bar plots and error bars indicate the mean ± SEM. **(C)** Results from a similar experiment in which, instead of perfusing dopamine, a steady holding current was applied, sufficient to depolarize the cell by ∼3 mV. Data are from a different sample of FS cells (*n* = 5) from the cells shown in **(B)**. **p < 0.01; *p ± 0.05; *ns*, not significant.

Two prominent types of interneurons were identified in layer 1a, horizontal (HZ) cells and neurogliaform (NG) cells (Figure 1A, right; Figure 1B). Consistent with previous findings (Suzuki and Bekkers, 2010), HZ cells had dendrites mainly projecting horizontally whereas NG cells had a smaller soma with dendrites branching in all directions (Figure 1B, top). HZ cells exhibited a biphasic AHP after each action potential while NG cells showed delayed onset to firing at rheobase (Figure 1B, bottom). In addition, the resting potential of HZ cells was more depolarized than that of NG cells (*HZ cells*, –72.4 ± 0.6 mV; *NG cells*, –80.5 ± 1.8 mV; *p* = 0.01, *n* = 5, unpaired *t*-test), while the input resistance was significantly lower in HZ cells (*HZ cells*, 39.9 ± 5.6 MΩ; *NG cells*, 79.2 ± 4.0 MΩ; *p* = 0.001, *n* = 5, unpaired *t*-test) and the rheobase was significantly greater in HZ cells (*HZ cells*, 696 ± 46 pA; *NG cells*, 398 ± 42 pA; *p* = 0.001, *n* = 5, unpaired *t*-test) (Figures 1E–G).

The two main classes of glutamatergic neurons in layer 2, semilunar (SL) cells and superficial pyramidal (SP) cells, could also be differentiated by their dendritic morphology and intrinsic electrical properties (Figure 1A, left; Figure 1C). Morphologically, SL and SP cells differed in the absence and presence, respectively, of basal dendrites (Figure 1C, top). The resting potential of SL cells was significantly more depolarized than that of SP cells (*SL cells*, –69.3 ± 1.2 mV; *SP cells*, –75.7 ± 0.8 mV; *p* = 0.003, *n* = 6, unpaired *t*-test) whereas the input resistance of SL cells was significantly higher than that of SP cells (*SL cells*, 145.1 ± 17.3 MΩ; *SP cells*, 73.9 ± 12.7 MΩ; *p* = 0.01, *n* = 6, unpaired *t*-test) (Figures 1E,F). The rheobase was not significantly different (*SL cells*, 133 ± 17 pA; *SP cells*, 223 ± 49 pA; *p* = 0.12, *n* = 6, unpaired *t*-test) (Figure 1G). All of these properties are consistent with earlier work (Suzuki and Bekkers, 2006).

In layer 3, two main types of interneurons with multipolar dendritic morphology were identified, regular-spiking (RS) cells and fast-spiking (FS) cells (Figure 1A, right; Figure 1D). The pattern of action potential firing was typically regular in RS cells and variable in FS cells (Figure 1D, bottom). Consistent with previous reports (Suzuki and Bekkers, 2010), the resting potential of RS cells was significantly more depolarized than that of FS cells (*RS cells*, –66.1 ± 1.5 mV; *FS cells*, –78.5 ± 1.4 mV; *p* = 0.0001, *n* = 7 and 8, respectively, unpaired *t*-test), the input resistance was significantly higher in RS cells (*RS cells*, 101.8 ± 4.0 MΩ; *FS cells*, 47.9 ± 4.3 MΩ; *p* = 0.002, *n* = 7 and 8, unpaired *t*-test) and the rheobase was significantly lower in RS cells (*RS cells*, 182 ± 61 pA; *FS cells*, 558 ± 85 pA; *p* = 0.006, *n* = 7 and 8, unpaired *t*-test) (Figure 1G). FS cells were also reliably identified by their expression of tdTomato in PV-Cre-tdTomato mice (data not shown), consistent with these being parvalbumin- (PV) expressing neurons.

The PCx contains additional classes of neurons, shown in gray in Figure 1A (Bekkers and Suzuki, 2013), namely, layer 2 bitufted (BT) interneurons, layer 2 and 3 NG cells, and layer 3 deep pyramidal (DP) cells and multipolar spiny (MS) cells. These neurons are relatively sparse (BT, layer 2/3 NG) or functionally uncharacterized (DP, MS) and were not studied here.

### Dopamine Depolarizes the Resting Potential, Increases the Input Resistance and Increases the Firing Frequency of Fast-Spiking Interneurons

Bath application of dopamine (DA, 10 μM) significantly depolarized the resting potential of FS cells in a reversible manner (Figures 2A–C; *control*, –78.7 ± 1.6 mV; *DA*, –75.1 ± 1.4 mV; *wash*, –80.6 ± 1.6 mV; *p* = 0.0001, *n* = 8 cells, repeated measures ANOVA using *lmer*). Dopamine also increased the input resistance of FS cells (Figures 2B,C; *control*, 49.7 ± 5.2 MΩ; *DA*, 59.7 ± 7.0 MΩ; *wash*, 57.0 ± 6.0 MΩ; *p* = 0.0006, *n* = 8 cells, *lmer*), but this effect was irreversible. In addition, dopamine caused a partially reversible increase in the frequency of firing of FS cells in response to a constant suprathreshold current step (Figures 2B,C; *control*, 9.1 ± 3.8 Hz; *DA*, 62.6 ± 8.7 Hz; *wash*, 42.2 ± 4.8 Hz; *p* = 0.0001, *n* = 8 cells, *lmer*). Intriguingly, the dopamine-induced increase in firing frequency seemed to persist for a longer period than the dopamine-induced depolarization of the resting potential (Figure 2B, right). Dopamine had no significant effects on the AHP amplitude, AP amplitude, AP voltage threshold and AP half-width of FS cells (data not shown).

Dopamine (10 μM) also increased the input resistance of HZ cells (Figure 2C, middle; *control*, 39.9 ± 6.3 MΩ; *DA*, 48.6 ± 5.6 MΩ; *p* = 0.024, *n* = 5 cells, *lmer*) and the firing frequency of SP cells (Figure 2C, right; *control*, 5.2 ± 0.8 Hz; *DA*, 9.9 ± 2.0 Hz; *p* = 0.024, *n* = 6 cells, *lmer*), but no significant effects were observed in any of the other intrinsic properties or cell types (Figure 2C). Thus, the remaining experiments focus solely on FS cells.

### Dopamine Modulation of Fast-Spiking Cells Is Functionally Similar to Injecting a Depolarizing Holding Current

In order to further quantify the effect of dopamine on the excitability of FS interneurons, we injected 1 s-long steps of depolarizing current of increasing amplitude into neurons under control conditions and in the presence of 10 μM dopamine (Figure 3A). The data were summarized as plots of AP firing frequency vs. amplitude of the injected current (f-I plots; Figure 3B, left). Dopamine caused a significant leftward shift of the f-I plot, quantified as a reduction in the rheobase (summary in Figure 3B, right, ‘Rheo’; *control*, 589 ± 75 pA; *DA*, 470 ± 85 pA; *p* = 0.0054, *n* = 5 cells, *lmer*). On the other hand, there was no significant effect of dopamine on the slope of the f-I plot (dashed lines, Figure 3B, left; summary in Figure 3B, right, ‘Slope’; *control*, 0.326 ± 0.023 Hz/pA; *DA*, 0.336 ± 0.033 Hz/pA; *p* = 0.72, *n* = 5, *lmer*).

These results are reminiscent of the effect of a steady depolarizing holding current on the f-I plot of many types of neurons (Silver, 2010). This was tested by making f-I plots for FS cells before and during application of a holding current that was sufficient to cause ∼3 mV depolarization of the resting potential, similar to that produced by dopamine (Figure 3C, left). As expected, application of holding current caused a significant reduction in rheobase with no significant change in slope of the f-I plot (Figure 3C, right; rheobase: *control*, 586 ± 172 pA; *holding*, 426 ± 130 pA; *p* = 0.025; slope: *control*, 0.395 ± 0.033 Hz/pA; *holding*, 0.402 ± 0.030 Hz/pA; *p* = 0.88, *n* = 5 cells, *lmer*). The change in rheobase caused by dopamine (119 ± 22 pA, *n* = 5) or by holding current (159 ± 45 pA, *n* = 5) was not significantly different (*p* = 0.46, unpaired *t*-test, *n* = 5) when a holding current of 89 ± 18 pA (range 49–138 pA) was applied. Thus, the effect of 10 μM dopamine on a FS cell is functionally similar to injecting about 90 pA of depolarizing current into the cell.

### The D1-Class Antagonist SCH 23390 Blocks Dopamine Modulation of Fast-Spiking Interneurons

**FIGURE 4:**
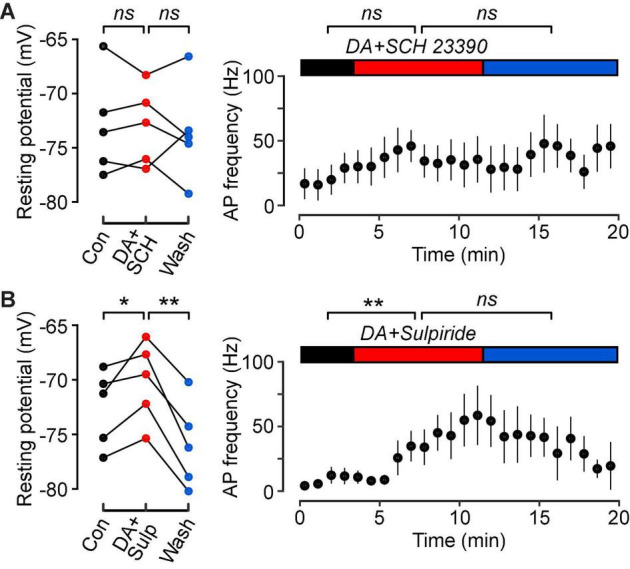
Dopamine D1-class receptors mediate the dopamine-induced increase in intrinsic excitability of FS cells. **(A)** (Left), Resting potentials of *n* = 5 FS cells in control (black), in 10 μM DA plus 10 μM of the D1-class antagonist, SCH 23390 (red), and wash (blue). Lines connect data points for the same cell. **(A)** (Right), Plot of mean action potential frequency vs. time for the same FS cells. Horizontal colored bars indicate the control, DA plus SCH 23390 and wash periods. SCH 23390 blocks the effect of dopamine on FS cell excitability. **(B)** Similar experiments with dopamine plus 10 μM of the D2-class antagonist, RS-sulpiride (*n* = 5 FS cells). RS-sulpiride does not block the DA-induced increase in excitability. **p < 0.01; *p < 0.05; *ns*, not significant.

In a final series of experiments, we aimed to elucidate the subtypes of dopamine receptors that mediate this modulation in FS cells. Co-application of dopamine (10 μM) with the D1-class dopamine receptor antagonist SCH 23390 (10 μM) prevented changes in the resting potential (Figure 4A, left; *control*, –72.9 ± 2.1 mV; *DA*, –73.0 ± 1.6 mV; *wash*, –73.6 ± 2.0 mV; *p* = 0.98, *n* = 5, *lmer*) and firing frequency (Figure 4A, right; *control*, 20.4 ± 11.4 Hz; *DA*, 33.6 ± 16.0 Hz; *wash*, 50.2 ± 15.7 Hz; *p* = 0.18, *n* = 5, *lmer*). However, there was still a small increase in input resistance following dopamine application and after washout (*control*, 42.5 ± 2.7 MΩ; *DA*, 45.5 ± 2.4 MΩ; *wash*, 49.7 ± 3.0 MΩ; *p* = 0.02, *n* = 5, *lmer*).

Co-application of dopamine with the D2-class dopamine receptor antagonist RS-sulpiride (10 μM) still resulted in a significant depolarization of the resting potential, and this effect was reversible (Figure 4B, left; *control*, –72.6 ± 1.6 mV; *DA*, –70.2 ± 1.7 mV; *wash*, –76.0 ± 1.8 mV; *p* = 0.04, *n* = 5, *lmer*). In addition, the input resistance increased (*control*, 38.9 ± 2.6 MΩ; *DA*, 43.0 ± 2.8 MΩ; *wash*, 44.3 ± 1.2 MΩ; *p* = 0.008, *n* = 5, *lmer*), as did the firing frequency (Figure 4B, right; *control*, 8.4 ± 3.4 Hz; *DA*, 50.4 ± 17.4 Hz; *wash*, 30.4 ± 9.5 Hz; *p* = 0.05, *n* = 5, *lmer*) during the co-application. However, these effects were irreversible following washout.

## Discussion

Previous research on the effects of dopamine in the PCx have focused on population activity (Collins et al., 1985; Gellman and Aghajanian, 1993; Schärer et al., 2012). Here, we have for the first time examined these effects at the level of individual neurons in the PCx.

We found that dopamine depolarized the resting potential, increased the input resistance and increased the firing of FS cells in response to a constant suprathreshold current step (Figures 2B,C). These results suggest that the increased spontaneous inhibitory activity in the PCx reported by Gellman and Aghajanian (1993) may be attributed to the increased intrinsic excitability of FS cells following dopamine application. We also found that the D1-class dopamine receptor antagonist SCH 23390 prevented dopamine modulation of FS cells (Figure 4A), while the D2-class dopamine receptor antagonist RS-sulpiride had no effect (Figure 4B). Our findings in the PCx are consistent with similar reports in the striatum (Bracci et al., 2002) and prefrontal cortex (Gorelova et al., 2002) implicating the D1-class receptors in dopamine neuromodulation of intrinsic excitability.

We also found effects of dopamine on the input resistance of HZ cells and the firing frequency of SP cells (Figure 2C). Because these effects were small and inconsistent across the three electrical parameters shown here (in contrast to FS cells), we regard them as tentative. We did not examine the effects of dopamine on deep pyramidal cells or multipolar spiny cells in layer 3, NG cells in layers 2 and 3, or bitufted interneurons in layer 2 (Bekkers and Suzuki, 2013). Given that we found minimal effects of dopamine in layer 2 glutamatergic neurons (Figure 2), it is anticipated that dopamine effects on layer 3 glutamatergic neurons will also be minimal. Similarly, we expect that NG cells in deeper layers, like those in layer 1a, will be unaffected by dopamine. Bitufted cells express vasoactive intestinal peptide (VIP) (Bekkers and Suzuki, 2013) and, to our knowledge, effects of dopamine on VIP-expressing neurons in other cortices have not been reported. Thus, we anticipate a similar lack of effect of dopamine on bitufted cells in the PCx. However, it would be important to test this hypothesis in future work.

Previous work on the zebrafish homolog of the PCx (Schärer et al., 2012) suggested that dopamine also reduces inhibitory synaptic transmission. Similar observations have been reported in the prefrontal cortex (Le Moine and Gaspar, 1998; Seamans et al., 2001; Trantham-Davidson et al., 2004). Although we did not examine inhibitory synaptic transmission, it is interesting to speculate that dopamine receptors may bidirectionally influence inhibitory activity in the PCx. Activation of D1-class receptors in the PCx increases the excitability of FS interneurons (this paper), whereas activation of D2-class receptors may decrease GABA-mediated synaptic currents (Schärer et al., 2012). Further experiments in the PCx could test this hypothesis.

Our observation that dopamine both depolarizes FS cells and increases the input resistance suggests that dopamine works by turning off an outward potassium current. However, we found no difference in the action potential half-width or the amplitude of the AHP that follows the action potential, ruling out the involvement of potassium currents which are responsible for swift repolarization following action potentials (Gruhn et al., 2005). Indeed, Gorelova et al. (2002) found that dopamine modulation of FS cells in the prefrontal cortex was independent of the delayed rectifier potassium current. Instead, Gorelova et al. (2002) found that dopamine suppressed three other types of potassium currents: the inactivating A-type potassium current, a potassium leak current and an inwardly rectifying potassium current. Suppression of each of these currents in turn resulted in the dopamine-induced increased firing frequency, depolarized resting potential and increased input resistance, respectively (Gorelova et al., 2002). Future work could explore the extent to which these findings from the prefrontal cortex also apply to the PCx.

What could be the consequences of dopamine-induced depolarization of FS cells for the operation of the PCx? As in other cerebral cortices, PV-expressing FS cells in the PCx are basket cells that powerfully inhibit the peri-somatic region of nearby excitatory neurons, suppressing action potential initiation (Stokes and Isaacson, 2010; Suzuki and Bekkers, 2012; Large et al., 2016). The function of FS cells in the PCx is still unclear but, by providing strong feedback and lateral inhibition, they may be important for establishing the sparse, distributed patterns of activity in excitatory neurons that are thought to encode odor identity (Stettler and Axel, 2009; Tantirigama et al., 2017; Bolding and Franks, 2018). In slices, FS cells have a relatively hyperpolarized resting potential and low input resistance, with the result that a substantial depolarizing current is required to reach AP threshold (i.e., high rheobase; Figures 1E–G). The depolarization caused by DA partially overcomes this reluctance of FS cells to spike (Figure 2C). Hence, DA may be critical for fine-tuning the recruitment of FS cells into the PCx circuit, with any perturbation in their activity likely to have major effects on the ability of the PCx to accurately discriminate odors.

The motor symptoms of Parkinson’s disease (PD), caused by a large reduction of dopaminergic neurons in the substantia nigra, can be preceded by a declining sense of smell that commences years earlier (Fullard et al., 2017; Schapira et al., 2017). Moessnang et al. (2011) found that human patients with early-stage PD exhibited significant hyperactivity of the PCx during an odor discrimination task. Additionally, Sancandi et al. (2018) explored the implications of reducing dopaminergic and noradrenergic inputs into the PCx in a PD mouse model. Following a substantial decrease of dopamine and noradrenaline innervation of the PCx, these authors found that neuroinflammation increased and the density of PV-expressing neurons significantly decreased. These structural changes were accompanied by olfactory impairments in multiple behavioral assessments (Sancandi et al., 2018). Thus, previous research suggests that depleted dopamine innervation in early PD leads to a reduced number of PV-expressing FS cells and hyperactivity of the PCx.

Our results, in conjunction with the key findings from previous studies, are consistent with the notion that a deteriorating sense of smell in early-stage PD is partly due to reduced dopaminergic innervation of the PCx and decreased density of FS cells. We suggest that these structural changes decrease inhibitory activity and lead to the hyperactivity of the PCx seen in early-stage PD. Thus, by elucidating the link between dopamine modulation and neuronal activity in the PCx, our results contribute to the growing understanding of how PD (and other neurodegenerative disorders) may impact olfactory regions of the brain and diminish the sense of smell.

## Conclusion

In conclusion, by using single-cell *in vitro* techniques, we have shown that dopamine acts on D1-class dopamine receptors on FS cells in the PCx to increase their intrinsic excitability. Our findings extend our understanding of how neuromodulators influence olfactory processing and provide a foundation for future investigations studying the role of FS interneurons in neurodegenerative disorders which are accompanied by olfactory impairments.

## Data Availability Statement

The raw data supporting the conclusions of this article will be made available by the authors, without undue reservation.

## Ethics Statement

The animal study was reviewed and approved by the Animal Experimentation Ethics Committee, The Australian National University.

## Author Contributions

YP performed the experiments and conducted the analysis. JB and YP developed the project, designed the experiments, and wrote the manuscript. Both authors contributed to the article and approved the submitted version.

## Conflict of Interest

The authors declare that the research was conducted in the absence of any commercial or financial relationships that could be construed as a potential conflict of interest.

## Publisher’s Note

All claims expressed in this article are solely those of the authors and do not necessarily represent those of their affiliated organizations, or those of the publisher, the editors and the reviewers. Any product that may be evaluated in this article, or claim that may be made by its manufacturer, is not guaranteed or endorsed by the publisher.
